# A demographic assessment of the impact of the war in the Gaza Strip on the mortality of children and their parents in 2023

**DOI:** 10.1186/s12963-025-00369-x

**Published:** 2025-03-03

**Authors:** Benjamin-Samuel Schlüter, Bruno Masquelier, Zeina Jamaluddine

**Affiliations:** 1 Digital and Computational Demography, Max Planck Institute for Demographic Research, Konrad-Zuse-Straße 1, 18057 Rostock, Mecklenburg-Vorpommern Germany; 2 Center for Demographic Research, UCLouvain, Place de l’Université, 1348 Louvain-la-Neuve, Belgium; 3 Department of Infectious Disease Epidemiology and International Health, London School of Hygiene and Tropical Medicine, Keppel Street, London, WC1E 7HT UK

**Keywords:** Gaza Strip, War, Mortality of children, Orphans, Prevalence of orphanhood

## Abstract

**Background:**

Following Hamas’s 7 October attack, Israel launched extensive aerial bombardments in the Gaza Strip, followed by a large-scale ground invasion. During the first 3 months of the conflict, up to December 31, 2023, the Palestinian Ministry of Health reported that 21,822 Palestinians were killed in Israeli strikes. This study estimates the number of excess deaths in children due to the war in the Gaza Strip in 2023 and assesses how the conflict has impacted the experience of parental loss among children.

**Methods:**

We reconstructed background life tables for the Gaza Strip based on under-five mortality estimates from sample surveys and accounted for casualties due to the 2023 conflict, using the age distribution of deaths from an individual list of 13,101 fatalities reported by the Palestinian Ministry of Health. We employed a kinship matrix model to estimate the number of new orphans in 2023 and the prevalence of maternal and paternal orphanhood.

**Results:**

From October 8 to December 31, 2023, our estimates indicate that 8120 children under 18 years of age were killed due to the conflict (with a range of 7099 to 9196 excess deaths). Additionally, 15,127 children (14,716–15,553) lost a father, and 9886 children (9564–10,216) lost a mother due to the conflict. Between 2022 and 2023, the probability of dying in childhood (ages 0–17) increased nearly sixfold for both males and females. The war increased the risk of losing a mother and a father by nine-fold and six-fold, respectively. Compared to the situation in 2022, the proportion of paternal orphans among children aged 0–17 rose by 1.5 times, while the proportion of maternal orphans doubled.

**Conclusions:**

The dramatic number of excess deaths among children and the sharp increases in orphanhood underscores the urgent need to prioritize the well-being and rights of children caught up in the war in Gaza.

**Supplementary Information:**

The online version contains supplementary material available at 10.1186/s12963-025-00369-x.

## Background

The day following Hamas’s October 7th attack, Israel launched airstrikes targeting the Gaza Strip, followed by a large-scale ground invasion. Since then, the extreme violence of the conflict, the scale of population displacement, the destruction of health infrastructure and housing and Israel’s blockade of the territory, cutting off residents from food, fuel, and water, have plunged the Gaza Strip into a humanitarian crisis of rare intensity [1–4]. The Palestinian Ministry of Health (MoH) in Gaza reported that 21,822 individuals were killed in the first 3 months of the conflict, up until December 31, 2023 [5]. This staggering death toll has continued to rise, with no end in sight at the time of writing this paper.

International humanitarian law requires all parties involved to take all necessary measures to protect civilians, particularly children. Children also have the right to be protected from conflicts under the United Nations (UN) Convention on the Rights of the Child [6]. And yet, in modern-day conflicts, the burden of losses generally falls heavily on civilian populations, with children and adolescents usually experiencing the highest burden of conflict-related deaths [7–9]. The conflict in Gaza is no exception: according to UNICEF, “Gaza has become a graveyard for thousands of children” [10]. Children also bear one of the most immediate consequences of conflicts, suffering the loss or separation from their parents. This reality was reflected by the emergence of the acronym “WCNSF” (wounded child, no surviving family) in November 2023 among medical care workers in Gaza, highlighting the tragic reality of children left without any family support [11].

Estimates of child deaths caused by conflicts and changes in the risk of becoming an orphan can help document the scale of a conflict from the perspective of children and enhance our understanding of the implications of a crisis for the affected populations [12–15]. These estimates can help determine whether the principles of international humanitarian law have been violated and reinforce efforts to hold political leaders and perpetrators accountable for crimes against humanity and war crimes in international courts [16]. However, these estimates are difficult to establish as they require granular information, such as age- and sex-disaggregated counts of deaths and the population at risk. Establishing the number of orphans also requires detailed age-specific patterns of fertility, for both mothers and fathers. Many conflicts occur in countries lacking comprehensive systems of death registration [8, 17], and when such systems are in place, they often collapse during severe crises.

In the context of the current conflict in the Gaza Strip, little is known about the crisis-related deaths beyond the death count produced regularly by the Ministry of Health in Gaza. The MoH figures have been regularly questioned, notably when the United Nations Office for the Coordination of Humanitarian Affairs (OCHA) published a breakdown of casualties in May 2024, categorizing deaths as formally identified, or missing or presumed under the rubble [18]. Yet the World Health Organisation (WHO), Israeli intelligence services, and the UN consider that the MoH estimates reflect the actual number of deaths [4]. The MoH death toll is also consistent with trends from deaths in staff members from the United Nations Relief and Works Agency for Palestine Refugees in the Near East (UNRWA) [19]. On October 26, 2023, the MOH published a comprehensive list of 6,747 victims, including their names, ages and identity numbers [20]. An examination of this list revealed no obvious reason to doubt the validity of the Ministry of Health’s data [3]. These data showed that children and young people (0–19) accounted for a significant fraction of deaths over this period (33.8%). A following extended list of 14,039 deaths with 13,101 having information on age and sex was released by the Ministry of Health on the 7th of January. This list encompasses casualties from the entire Gaza Strip from November 2, 2023, until January 5, 2024 [21].

There are significant uncertainties regarding the total number of children who died in 2023, although some figures have been reported in the press. The MoH suggested that “more than 10,000 children” had been killed in the first 3 months of the conflict in 2023 due to airstrikes and ground operations in Gaza [22]. In addition to the deaths of children, several estimates of the number of children who have lost their parents have also circulated in the press. UN Women estimated that more than 10,000 children may have lost their fathers due to the conflict in 2023 [23]. The Euro-Med Human Rights Monitor estimated that 24,000 to 25,000 Palestinian children had lost one or both parents in 2023 as a result of the war [24].

In this study, we first reconstruct life tables for the period pre-conflict in the Gaza Strip. We then use age-sex average relative risks of mortality observed in the initial days of the war to disaggregate the total number of deaths from the MoH and estimate the number of children that might have died due to the conflict in 2023. Additionally, after reconstructing past trends in fertility, we use a kinship matrix model [25] to assess how the conflict has impacted the risk of orphanhood for children. We limit the study to the first 3 months of the conflict to obtain annual mortality and orphanhood estimates, enabling comparison with the situation in 2022. Another reason for focusing on 2023 is the potential decline in the quality of MoH mortality data as the war progressed, due to the destruction of infrastructure, including health facilities. The official death tolls were likely less complete in 2024 than in 2023, as suggested by the increasing proportion of unidentified deceased persons [4] (Table 1).

## Methods

### Reconstruction of background mortality and fertility trends in the Gaza Strip

Demographic estimates provided by international agencies such as the United Nations Population Division and UNICEF typically refer to the entire State of Palestine [26, 27]. To focus specifically on Gaza, we gathered data from multiple data sources.

**Table 1:** Data sources used in this study

Data source	Description	Information extracted for this study	Strengths	Limitations
International Database from the U.S. Census Bureau	Database of population by age and sex (and demographic characteristics) for over 200 countries and areas, with subnational breakdown	Age and sex distribution of the population in the Gaza Strip	Widely used by multiple agencies and researchers; employs well-established tools for demographic analysis	Data sources and adjustments for the Gaza Strip are not fully documented. Lack of recent input data (last census in 2017)
Population estimates from the Palestinian Central Bureau of Statistics (PCBS)	Mid-year population estimates and projections for each Governorate in Palestine from 1997 to 2026	Total population for the Gaza Strip from 1997 to 2023	Based on rigorous demographic techniques and local expertise, combining census data, vital registration and household surveys. Widely used by international organizations and development partners	Trends until 2017 based on successive censuses, but more recent extrapolations less precise due to the incompleteness of birth and death registration
Multiple Indicator Cluster Surveys (2000, 2010, 2014 and 2019–2020)	Multi-stage cluster sample surveys carried out by the PCBS with the technical and financial support of UNICEF to collect data on indicators related to the health, education, and well-being of women and children.	Under-five mortality rates extracted from birth histories in the microdata or from tabulations, age-specific fertility rates in reports	Standardized sampling techniques and questionnaires, extensive pre-tests, high response rate	Survey implementation and sample representativeness possibly affected by the recurrent conflicts
Other country-specific surveys	1995 and 2004 Demographic Surveys, 2006 Palestinian Family Health Survey	Under-five mortality rates and age-specific fertility rates in published reports	Representative at the regional level, high response rate	Less standardized than the MICS surveys
Number of deaths reported by the Palestinian MoH	Conflict-related fatalities documented by the Palestinian MoH based on hospital reports in Gaza	Total number of deaths due to the war from October 7h to December 31, 2023 (21,822), and detailed list of 13,101 fatalities with age and sex information	Historically considered as accurate in previous conflicts, and consistent with other sources of mortality reporting in the first months of the recent conflict [19]	Possible underreporting of deaths due to challenges in mortality verification, such as bodies trapped under rubble or infrastructure damages [4]
Child mortality database from the UN Inter-Agency Group for Child Mortality Estimation	Database of globally standardized estimates of neonatal, infant and under-five mortality rates	Sex-specific estimates of under-five mortality in the State of Palestine from 1997 to 2021	Based on a comprehensive analysis of surveys, censuses and vital registration data, with adjustments for biases in data series and quantification of uncertainty [28]	Possible biases due to sampling and non-sampling errors. Trends for recent years (2016–2021) in the State of Palestine based on extrapolation from past surveys and censuses

First, we extracted age- and sex-specific population estimates for the Gaza Strip from the US Census Bureau International Database [29]. These estimates were re-scaled to align with the mid-year population counts available from the Palestinian Central Bureau of Statistics (PCBS) for the Gaza Strip spanning from 1997 to 2023.

**Fig. 1:**
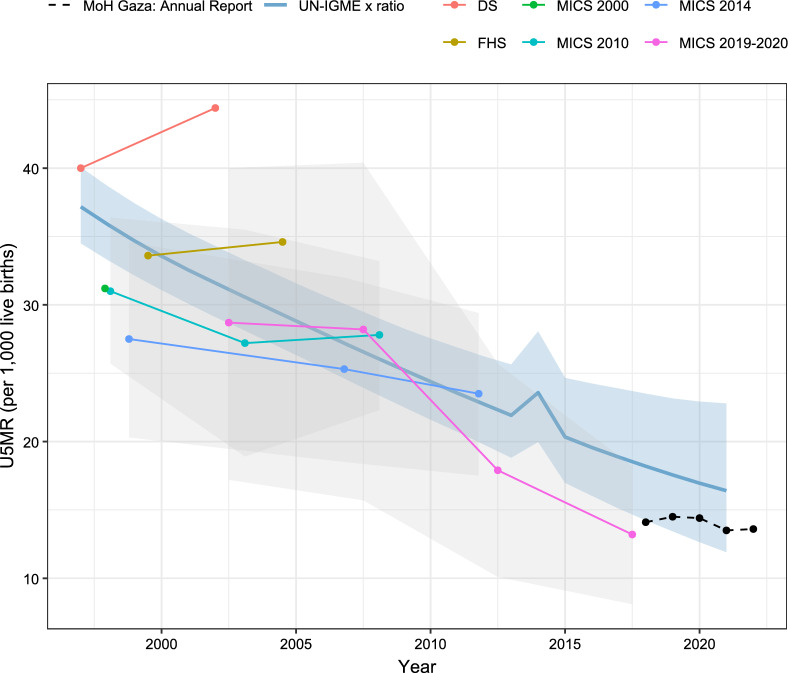
Trend in the under-five mortality rate obtained by scaling up the UN IGME estimate for the State of Palestine, compared to estimates from surveys and annual reports of the Ministry of Health for the Gaza Strip

Second, to reconstruct past trends in mortality, we extracted under-five mortality rates (U5MR) for both the entire State of Palestine and specifically for the Gaza Strip from previous surveys. We recalculated U5MR for three 5-year periods using full birth histories in the Multiple Indicator Cluster Surveys (MICS) conducted in 2010, 2014 and 2019–2020 [30–32]. We also extracted estimates from published reports from the Palestinian Family Health Survey 2006, the 2004 Demographic Survey, and the 2000 MICS [33–35]. From these sources, we computed the mean ratio of the U5MR in the Gaza Strip to the U5MR in the State of Palestine, for all estimates falling in the period 1997–2023, yielding a mean ratio of 1.11. This mean ratio was then applied to the sex-specific estimates of U5MR for the State of Palestine from 1997–2021, as provided by the UN Inter-Agency Group for Child Mortality Estimation (UN IGME) [27]. To estimate U5MR for 2023, we extrapolated the time series for each sex separately, assuming an exponential decline and using the period 2015–2021 as a reference. The resulting trend in U5MR is presented in Fig. 1, alongside estimates from sample surveys conducted in Gaza, and recent estimates from vital registration available from the Gaza Health Ministry. To generate a full life table, we combined the trend in U5MR with standard age patterns of mortality, representing expected relationships between mortality in children under 5 and all other age groups. We retained the West pattern of the Princeton model life tables [36], as used by the United Nations Population Division for the State of Palestine [26]. The resulting background life tables refer to the Gaza Strip population from 1997 to 2023, as if the most recent conflict had not occurred.

**Fig. 2:**
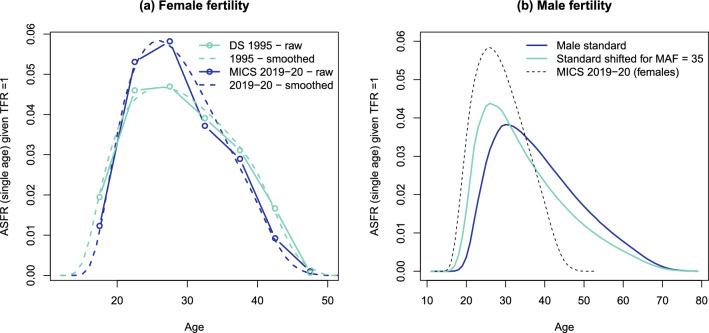
**a** Age-specific fertility rates for females (given total fertility rate= 1) from the 1995 and 2019–2020 surveys, and smoothed rates, **b** Standard age pattern of fertility rates for males, standard shifted for mean age at fatherhood (MAF) of 35 years, compared to smoothed rates for females for 2019–2020

Third, we reconstructed the trend in age-specific fertility rates (ASFR) to calculate the probability of becoming an orphan. ASFR estimates for Gaza were extracted from published reports of five surveys: the 1995 Demographic Survey, 2004 Demographic and Health Survey (DHS), 2006 Family Health Survey, and MICS conducted in 2000, 2014 and 2019–2020. While these ASFRs were available for 5-year age groups for specific periods, our model required single-year estimates for all calendar years. We smoothed fertility rates using the relational Gompertz method and extracted two parameters from each survey: $\alpha$, which captures the age pattern of the fertility schedule, and $\beta$, which captures the spread of the fertility schedule [37]. The smoothing is illustrated in Fig. 2a for the 1995 demographic survey and the 2019–2020 MICS survey, using a fertility standard developed by Booth [38]. Assuming a linear trend in the $\alpha$ and $\beta$ parameters, we then reconstructed the set of rates by single years of age for each calendar year between 1997 and 2023.

Age-specific fertility rates for males are also required for calculating the proportions of paternal orphans, yet this information is often unavailable in surveys. We used a standard pattern, developed by Paget and Timaeus [39], reflecting higher ages at the onset of fertility and a larger spread than for female fertility (Fig. 2b). We adjusted this standard to align with a mean age at fatherhood of 35 years, as estimated by Schoumaker (2019) [40] for the State of Palestine.

### Combination of war-related deaths and background life tables

The number of deaths between October 7th and December 31st was retrieved from the MoH (21,822 deaths) [5]. We assumed that this death toll consists of additional deaths that would not have occurred had the war not started. Deaths due to the war are thus considered as an additional cause of death. Our estimates are conservative as we did not consider the additional deaths that might have occurred due to the breakdown of the health care system, destruction of housing and increased incidence of infectious diseases resulting from overcrowding, bad sanitation and malnutrition [41].

As indicated earlier, we had access to a list released in January 7, allowing us to isolate 13,101 deaths that occurred between October 7 and December 31 with individual information on each death. We used information on the dates of birth and death, and sex of the deceased to compute the distribution of war-related deaths by sex and age. We assumed that this age- and sex-specific death distribution reflects the distribution of all deaths due to the war before December 31st and used it to redistribute the 21,822 deaths by age and sex. To reduce the noise and potential age heaping, we aggregated death counts into 5-year age bins (0–4, 5–9,..., 75+) (see Supplementary Figure S1, “Supplementary Information”). To match the age granularity from the life table (1-year age groups until 95+ years old), we ungrouped the death counts with the method proposed by Rizzi et al. [42] (see Supplementary Figure S2, “Supplementary Information”). The death counts for single years of age were then combined with population exposures to compute sex- and age-specific mortality rates due to the war.

### Calculation of the probability of losing a parent

The kinship matrix model [25, 43] uses fertility and mortality rates specific to age, sex and year to estimate implied populations of surviving parents as seen from the perspective of children (by age). These populations are projected forward in time, incorporating time-varying survival probabilities. The projection model is estimated separately for both males and females. Mathematically, the model allows to project the population of parents of a child according to the equations that follow (simplified by showing the model for one sex only),

1 $$\begin{aligned} \begin{pmatrix} \varvec{d}_{Living} \\ \hline \varvec{d}_{Dead} \end{pmatrix}(x+1, t+1)&= \left( \begin{array}{c|c} \varvec{U}_t & \varvec{0} \\ \hline \varvec{M}_t & \varvec{0} \\ \end{array}\right) \begin{pmatrix} \varvec{d}_{Living} \\ \hline \varvec{d}_{Dead} \\ \end{pmatrix}(x, t) \end{aligned}$$

2 $$\begin{aligned} \begin{pmatrix} \varvec{d}_{Living} \\ \hline \varvec{d}_{Dead} \end{pmatrix}(0, t)&= \begin{pmatrix} \frac{\varvec{f_x} \circ \varvec{n_x}}{||\varvec{f_x} \circ \varvec{n_x}||} \\ \hline \varvec{0} \end{pmatrix} \end{aligned}$$

where $\varvec{d}_{Living}(x,t)$ is a vector of dimension ($\omega \times 1$) reflecting the population of parents alive at age *x* of a child in year *t*, $\omega =96$ is the upper age bound considered, $\varvec{d}_{Dead}(x,t)$ is a vector of dimension ($\omega \times 1$) reflecting the population of dead parents at age *x* of a child in year *t*, $\varvec{U_t}$ is a matrix of dimension ($\omega \times \omega$) containing the survival probabilities on its main subdiagonal, $\varvec{M_t}$ is a matrix of dimension ($\omega \times \omega$) containing the probabilities of dying on its main diagonal, $\varvec{f_x}$ is a vector of dimension ($\omega \times 1$) containing the age-specific fertility rates, and $\varvec{n_x}$ is a vector of dimension ($\omega \times 1$) containing the age-specific population counts. The model makes two key assumptions. First, it assumes that a parent is alive at the birth of its child ($\varvec{d}_{Dead}(x,t)=\varvec{0}$ at $x=0$ in Eq. 2), which is obvious for mothers but not for fathers who may die between conception and birth. Second, the age distribution of parents at birth of a child is a function of the fertility rates and the population age structure ($\varvec{d}_{Living}(x,t)=\frac{\varvec{f_x} \circ \varvec{n_x}}{||\varvec{f_x} \circ \varvec{n_x}||}$ at $x=0$ in Eq. 2). The age schedule of fertility rates is thus determining the shape of the age distribution of parents. It is this age distribution of parents that is then projected forward in time by applying the time-varying survival probabilities. Our model assumes that the population is stable before 1997 (see Supplementary Section 2, “Supplementary Information”).

The probability of experiencing the death of a parent is the ratio between the expected number of parents dying at age *x* of a child and the expected number of parents alive at age *x* of a child. In year *t* and for a child age *x*, it is expressed as

3 $$\begin{aligned} p(x,t) = \frac{||\varvec{d}_{Dead} (x,t)||}{||\varvec{d}_{Living} (x,t)||}. \end{aligned}$$

To compute the number of children under the age of 18 years who could have lost a parent at the population level, we need to weight that probability according to the population age structure of the Gaza Strip population as follows:

4 $$\begin{aligned} {\text {Number of children under 18 years old losing a parent}} = \sum _{x=0}^{18} p(x,t) \cdot n_x(t). \end{aligned}$$

Assessing the impact of the war on the probability of losing a parent in the year 2023 can be done by augmenting $\varvec{M_t}$ into a matrix of dimensions ($2\omega \times \omega$). The augmented matrix allows us to differentiate between the probability of dying that would have been observed, had the war not happened, and the probability of dying due to the war. The background mortality schedule in the year 2023 was taken as the mortality level that would have been observed without the war. The probabilities of dying due to the war were computed using the mortality rates associated with the war.

Since the kinship matrix model is deterministic, the model outputs are not associated with uncertainty measures. To incorporate uncertainty into our estimates, we performed the projection within a Monte-Carlo simulation, running the projection over 2000 iterations. At each iteration, uncertainty was incorporated through two elements: 1) uncertainty in the background U5MR estimates from the UN IGME and 2) stochasticity from the additional deaths due to the war. More precisely, at each iteration, we drew a U5MR estimate, assuming they are normally distributed with the bounds provided by UN IGME. We then generated full life tables from the obtained U5MRs, using the West pattern of Princeton model life tables [36] (see Supplementary Figure S3, Supplementary Information). We also reflected the stochasticity in war deaths by assuming that these deaths are Poisson distributed, and we generated age- and sex-specific death counts at each iteration. The death counts were then converted into mortality rates due to the war using the population exposures.

### Comparison of patterns of excess mortality and orphanhood from other sources

Because we only have access to an age and sex distribution for two-thirds of war-related deaths in 2023 (13101/21822), we compared this distribution with age distributions for other conflicts to evaluate if it is plausible. To improve the estimation of excess child mortality in crisis settings, Mathers et al. [17] assembled a comprehensive database of age-sex distributions of deaths according to nine categories of crisis events (conflicts, conflicts for combatants, genocides, earthquakes, tsunamis, cyclones, floods, epidemics, and famines). They used data from 164 crises in 57 countries. Using this database, they estimated average relative risks of mortality by age and sex, for each event type. The UN IGME uses these risks to redistribute excess crisis deaths by age when estimating under-five mortality [28]. We compared these average relative risks of mortality by age and sex to the ones computed using the age-sex-specific death distribution from the MoH. We considered three types of crisis events: conflict, earthquake, and genocide. Mathers et al. [17] estimated these crisis events based on n = 35, n = 27, and n = 4 sources (studies, surveys, or death registration), respectively. We considered the pattern associated with earthquakes as these large-scale disasters cause widespread damage, which can reflect the impact on mortality of infrastructure collapse following airstrikes. The pattern associated with genocide is considered as it is quite different from the other two, with more widespread excess mortality. In addition, in January 2024, the International Court of Justice ruled that there was a credible risk that irreparable prejudice would be caused to the rights of Palestinians in Gaza under the genocide convention.^1^

We also compared our estimates of orphanhood with data from the Palestinian MoH. On May, 1st, 2024, the MoH released a list of 15,173 children aged 0–17 who had lost one or both parents due to the war [44]. This list was assembled by looking for children in the Palestinian civil registry born to the identified adults who had died due to the war. This list gives only a partial view of the orphan situation, as only children whose parents are identified in the MoH lists can themselves be identified. The list does, however, provide a lower bound for our estimates and an age breakdown which can be compared to our estimates: 25% of the orphans on this list were under 5, 32% were between 5 and 9, 29% between 10 and 14 and 14% between 15 and 17. In terms of sex distribution, 48% of these children were female, and 52% were male. This list also provides an estimate of the number of orphans who have lost both parents: of the 15,173 orphans under 18 years old, 911 had lost both parents, i.e. 6%. We used this proportion to deduce the total number of orphans after calculating maternal and paternal orphans separately.

## Results

### Age-sex distribution of deaths due to the war

**Fig. 3:**
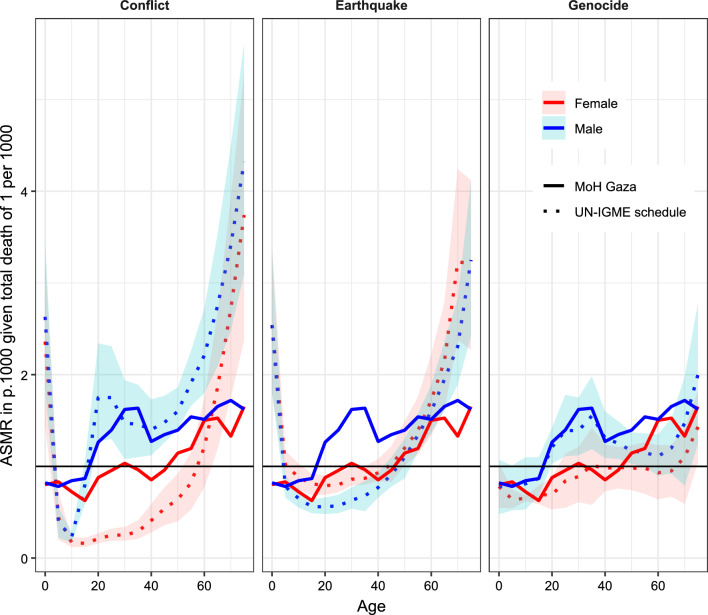
Age-specific death rates given total crisis death rate of 1 per 1000 population, comparing three types of crisis events with the age distribution in the list of identified casualties

Figure 3 compares the age-specific death rates given the total crisis death rate between three UN IGME standard patterns (conflict, earthquake, and genocide) with the one obtained using the death distribution of the latest deaths list shared by the Ministry of Health in Gaza. The mortality from the current war in Gaza does not exhibit strong differences between males and females, distinguishing it from the conflict schedule. The age pattern associated with earthquakes shows a higher burden on young children and older adults than what is observed during the war in Gaza. The age pattern associated with genocide is the closest to the observed death rates due to the war, especially below the age of 40 years.

### Deaths in children and under-five mortality

**Fig. 4:**
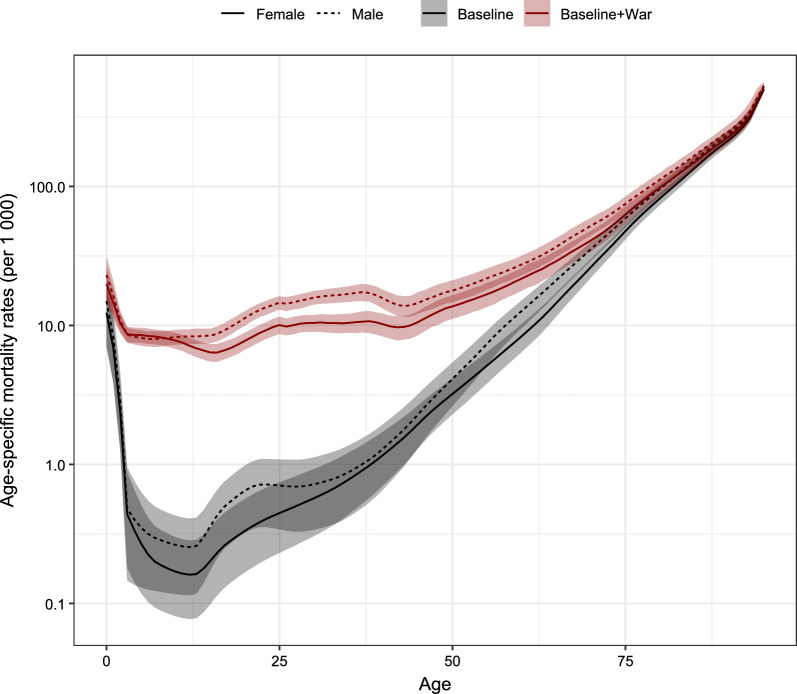
Sex- and age-specific mortality rates (and 95% CI) in the absence of the 2023 war (black lines) and accounting for the 2023 war (red lines) in the Gaza Strip

Figure 4 shows, using a log scale, the sex- and age-specific mortality rates in 2023 obtained by converting the overall war mortality rates using the age- and sex-specific death distribution from the Palestinian MoH. We also show the expected 2023 mortality age patterns had the war not happened (black lines).

We computed the life expectancy at birth in two cases: in the absence of the war, and adding the excess mortality due to the war. Our estimates suggest that life expectancy for males dropped by 24 years (95% CI 22.6–25.8) because of the conflict, from 73.2 (95% CI 70.3–76.8) to 49.2 (95% CI 47.7–51.0) years in 2023 (Table 2). For females, it dropped by 21.3 years (95% CI 20.2–22.4), decreasing from 76.1 (95% CI 73.4–78.7) to 54.8 (95% CI 53.2–56.3) years. The U5MR ($_{5}q_{0}$) increased by close to three-fold for females and males when we account for the effect of the war in 2023. For children and youth less than 18 years old, the probability of dying ($_{18}q_{0}$) increased by close to six-fold for both females and males.

**Table 2:** Life expectancy at birth ($e_{0}$), and probabilities of dying for children aged less than 5 ($_{5}q_{0}$) and less than 18 ($_{18}q_{0}$) for the Gaza Strip, in the absence of the 2023 war and with war-related deaths added

		$e_{0}$	$_{5}q_{0}$	$_{18}q_{0}$
		**Females**		
**2022**	Estimates	76.0 (73.4–78.7)	0.022 (0.012–0.034)	0.025 (0.014–0.039)
**2023**	War-free	76.1 (73.4–78.7)	0.022 (0.012–0.034)	0.025 (0.014–0.039)
	War	54.8 (53.2–56.3)	0.060 (0.050–0.072)	0.146 (0.136–0.159)
		**Males**		
**2022**	Estimates	73.1 (70.2–76.7)	0.027 (0.013–0.042)	0.031 (0.015–0.048)
**2023**	War-free	73.2 (70.3–76.8)	0.026 (0.013–0.041)	0.030 (0.014–0.048)
	War	49.2 (47.7–51.0)	0.064 (0.052–0.079)	0.161 (0.146–0.176)

**Fig. 5:**
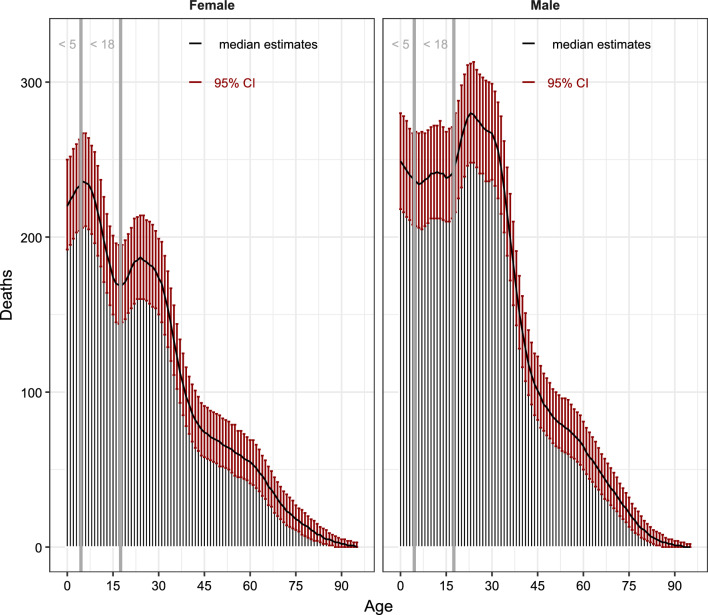
Sex and age distribution of deaths due to the 2023 war in the Gaza Strip

Figure 5 shows the estimated number of deaths by age, and sex (left/right panels) due to the war. On the left of the vertical grey lines are the children below 5 and 18 years old, respectively. Among children aged less than 5 and between 5 and 18 years old, we estimate that there were 2351 (95% CI 2059–2652) and 5769 (95% CI 5040–6544) deaths due to the war, respectively. In total, we estimate that 8120 children under 18 years of age were killed due to the war (95% CI 7099–9196) in 2023. As much as 11% of all war-related deaths were among children under 5 years old (95% CI 9–12) and 37% of all war-related deaths were among children under 18 years old (95% CI 33–42).

### Parental loss

The share of children that would have experienced the death of a parent in 2023, had the war not happened, is 0.11% (95% CI 0.07–0.16) and 0.26% (95% CI 0.17–0.34) for mothers and fathers, respectively. We estimate that because of the war, an additional 0.95% of children lost their mother (95% CI 0.92–0.98) and 1.45% of children lost their father (95% CI 1.41–1.49) in 2023. This translates into 9886 (95% CI 9564–10,216) and 15,127 (95% CI 14,716–15,553) new maternal and paternal orphans due to the war in the first 3 months of the conflict. Out of these orphans, we estimate that 28% are under 5 years old. This proportion is consistent with the list of orphans released by the Palestinian MoH (25%). Using the proportion of double orphans among all orphans reported in this list (6%), we calculate that the war created 1,416 (95% CI 1374–1459) double orphans. This translates into 23,597 (95% CI 22,906–24,310) children that have lost one or both parents due to the war, in the first 3 months of the conflict.

**Fig. 6:**
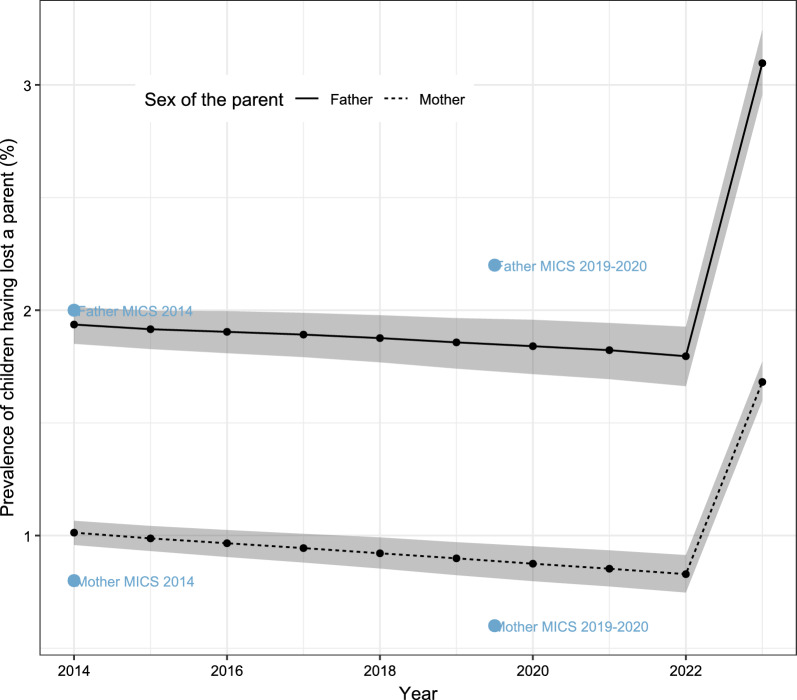
Prevalence of maternal and paternal orphanhood over the period 2014–2023 as estimated from our model and reported in the 2014 and 2019–2020 MICS surveys

The numbers above refer to the incidence of orphanhood (that is, the proportion of children who lost at least one parent over the course of a year). The incidence can also be converted into the prevalence of orphanhood (the proportion of children aged 0–17 in 2023 who have lost at least one parent in their lifetime). Figure 6 shows the prevalence of maternal/paternal orphanhood over the period 2014–2023. The prevalence of paternal orphanhood was around 2% and according to our estimates, it had declined slightly over the period 2014–2022. The maternal orphanhood prevalence was lower, around 1% for mothers, due to lower female mortality and younger ages at childbearing. It had also declined during the years 2014–2022. Figure 6 shows that our model estimates are generally close to the survey estimates for Gaza from the 2014 and 2019–2020 MICS for fathers, but they are higher than the surveys for mothers. This could be due to misreporting in the surveys due to the adoption bias, a pattern observed elsewhere and deemed to be more frequent for maternal orphans [45]. When considering only the model-based estimates, the 2023 war increased the estimated prevalence of paternal orphans to 3.10% (95% CI 2.96–3.24), while the prevalence of maternal orphanhood reached 1.68% (95% CI 1.60–1.77).

## Discussion and conclusions

In this study, we aimed to assess the impact of the war in the year 2023 on the mortality of children and their parents. We did this by reconstructing the mortality and fertility rates by age and sex for the Gaza Strip over the period 1997–2023. We measured the additional mortality burden caused by the war by redistributing the 21,822 deaths reported by the Palestinian MoH using the age- and sex-specific death distribution from a list of 13,101 deaths that could be identified up to December 31st, and converted them into mortality rates due to the war. We used these rates as inputs in a kinship matrix model to calculate the number of orphans in 2023 due to the war.

The war significantly increased the mortality experienced by the population of the Gaza Strip, as testified by the stark drop of 21.3 and 24.0 years of life expectancy for females and males, respectively. Of particular concern is the similitude between the age schedule of relative risks in the first 3 months of the current war and the UN IGME schedule of excess deaths reflecting genocide. This highlights the importance of the International Court of Justice’s request for the indication of measures from Israel to fulfil their obligations under the Convention on the Prevention and Punishment of the Crime of Genocide [46].

Children have been severely affected by the conflict. According to our analysis, as many as 8,120 (95% CI 7099–9196) children under the age of 18 years old may have lost their lives due to the war in 2023. Between 2022 and 2023, the probability of dying in this age group increased by close to six-fold for females and males. Out of the children surviving the war, 15,127 (95% CI 14,716–15,553) and 9886 (95% CI 9564–10,216) may have lost a father and a mother due to the conflict, respectively. In total, assuming that 6% of orphans were double orphans, 23,597 (95% CI 22,906–24,310) children have lost one or both parents. Our estimates are consistent with those of UN Women, which stated that more than 10,000 children may have lost their fathers due to the conflict (UN Women 2024). They are only slightly lower than the estimate from the Euro-Med Human Rights Monitor, which suggested that 24,000–25,000 children had lost one or both parents as a result of the war. Our estimates are, however, significantly higher than the list of 15,173 orphans released in May by the Palestinian MoH in Gaza. The MoH’s estimation of orphans was based on identified deaths that could be linked to civil registration records. This methodology likely resulted in the under-reporting of orphans as it excluded cases where parents’ deaths were not formally recorded or where identification was impossible, and it omitted newborns who had not been registered in the civil registry due to the ongoing conflict. At that time, the MoH had documented the identities of 24,682 out of 34,654 reported casualties, leaving 29% of casualties without names or identification numbers.

Our analysis has important limitations that should be taken into account when interpreting the results. First, we did not account for migration when reconstructing the population dynamics of the Gaza Strip. However, due to the strict control at the borders, the migration flows were relatively small compared to the overall population. According to the Palestinian ambassador in Egypt, 80,000–100,000 have left Gaza between the beginning of the war and April 2024 [47]. Failure to account for out-migration might nevertheless lead to underestimating mortality rates due to the war by depleting the population at risk. Second, we only focused on the first 3 months of the war (until December 31st), to be able to compare annual changes in life expectancy and age-specific mortality, and to reduce the biases associated with the missing data on age and sex for a growing share of deaths and the higher likelihood of under-reporting of deaths by the Palestinian MoH in Gaza due to the collapse of infrastructures [4]. Additionally, the age- and sex-specific death distribution during the earlier stages of the war might not reflect the future developments of the conflict. For example, women and children of Gaza have been shown to be killed less frequently as the war’s toll rises [48]. Third, we measured the mortality associated with the war with data collected from the Palestinian MoH in Gaza. Due to the ongoing crisis, this source might underestimate the true number of direct deaths. Fourth, it is difficult to disentangle the direct and indirect impact of the war on mortality. Again, focusing on the first 3 months allowed us to assume that we mostly captured the direct effect of the war as the conflict was in its earlier stage. However, insofar as the war went on for more than a year, with no end in sight at the time of writing, many more deaths are to be expected because of malnutrition, improper sanitation, no access to health care systems and causes such as reproductive, communicable, and non-communicable diseases [1–4]. Fifth, to estimate the incidence and prevalence of paternal orphanhood, we had to reconstruct the fertility age schedule based on a standard age pattern and the mean age at fatherhood. We assumed that the male ASFRs were constant over the period studied. This has a limited impact on our estimates as the kinship matrix model only requires capturing the age pattern of fertility, but not its level, which might have decreased over 1997–2023. Finally, according to the health ministry, deaths are highly concentrated in some families. For example, between October 7 and 31, at least 6120 fatalities were from 926 families, with 444 families losing 2–5 members, 136 families losing 6–9 members, and 192 families losing 10 or more members [20]. This clustering of deaths within families is not accounted for in our model and could lead to overestimating the number of orphans still alive. When analyzing the list of 15,173 orphaned children provided by the MoH, we could only identify 103 children whose names also appeared in the list of casualties, but more work is needed for a more comprehensive assessment of the excess mortality of orphans. Because of these various limitations, our estimates should be considered with caution. Whenever this becomes feasible when field conditions improve, retrospective mortality surveys should be conducted to document more precisely the impact of the war on mortality and orphanhood.

While conservative and covering the first 3 months of the war, our estimates show a dramatic effect of the war on child lives and safety, with long-lasting effects across several generations to be feared [1, 2]. Premature deaths of children and parents are the most visible manifestations of severe violations of children’s rights. All children in Gaza have endured the traumatic realities of conflict, with the vast majority having been forcibly displaced. Children are further affected by the destruction of schools and healthcare facilities, which is considered by the UN a grave violation against children during armed conflict [6]. Denial of humanitarian access, another grave violation, means that many children now face catastrophic levels of malnutrition [49, 50], and have no adequate provision of clean water, sanitation and shelter. Several hundred children requiring urgent medical evacuation remain trapped in Gaza.^2^

We hope that our estimates can reinforce the calls made by multiple organizations for an immediate and lasting humanitarian ceasefire. A safe corridor out of Gaza for children with life-threatening conditions (and their families) should be urgently established. Measures are also required to ensure that civilian infrastructures such as schools and health facilities are rehabilitated. Emergency humanitarian aid should be deployed to meet the most pressing needs of the affected populations. In the longer term, social support will need to be provided to all orphans and affected children, to mitigate the effects of these traumatic life experiences on their health and development.

## Supplementary Information

Supplementary Material 1.

## Data Availability

The datasets supporting this study are publicly available. An updated series of datasets on known victims is available here: https://data.techforpalestine.org/docs/datasets/.

## Abbreviations

ASFR: Age-specific fertility rates
DHS: Demographic and Health Survey
MICS: Multiple Indicator Cluster Surveys
MoH: Palestinian Ministry of Health in Gaza
OCHA: Office for the Coordination of Humanitarian Affairs
PCBS: Palestinian Central Bureau of Statistics
U5MR: Under-five mortality rates
UN: United Nations
UNICEF: United Nations Children’s Fund
UN IGME: United Nations Inter-Agency Group for Child Mortality Estimation
UNRWA: United Nations Relief and Works Agency for Palestine Refugees in the Near East
WCNSF: Wounded child, no surviving family
WHO: World Health Organisation

## Footnotes

1 In November 2024, the Pre-Trial Chamber I of the International Criminal Court also found reasonable grounds to believe that Mr Netanyahu (Prime Minister of Israel) and Mr Gallant (Minister of Defence of Israel at that time) “bear criminal responsibility for the war crime of starvation as a method of warfare”. The Chamber also found there are “reasonable grounds to believe that the lack of food, water, electricity and fuel, and specific medical supplies, created conditions of life calculated to bring about the destruction of part of the civilian population in Gaza”. https://www.icc-cpi.int/news/situation-state-palestine-icc-pre-trial-chamber-i-rejects-state-israels-challenges.

2 https://www.unicef.org/sop/press-releases/dying-waiting-gazas-children-face-lethal-delays-medical-evacuation.
