# Correction to: A demographic assessment of the impact of the war in the Gaza Strip on the mortality of children and their parents in 2023

**DOI:** 10.1186/s12963-025-00375-z

**Published:** 2025-03-31

**Authors:** Benjamin-Samuel Schlüter, Bruno Masquelier, Zeina Jamaluddine

**Affiliations:** 1 Digital and Computational Demography, Max Planck Institute for Demographic Research, Konrad‑Zuse‑Strase 1, 18057 Rostock, Mecklenburg‑Vorpommern, Germany; 2 Center for Demographic Research, UCLouvain, Place de l’Universite, 1348 Louvain‑la‑Neuve, Belgium; 3 Department of Infectious Disease Epidemiology and International Health, London School of Hygiene and Tropical Medicine, Keppel Street, London, WC1E 7HT UK

**Correction to:** ***Popul Health Metrics 23, 8 (2025)***

10.1186/s12963-025-00369-x

The original publication of this article contained 2 errors:

- The author names were inverted.
- Equation 4 contained erroneous “\;” symbols in the online version.

The correct author names are shown in this correction article, the original article has been updated.

