# Supplementary material for: A demographic assessment of the impact of the war in the Gaza Strip on the mortality of children and their parents in 2023
Source: Popul Health Metr. 2025 Mar 3;23:8. doi: 10.1186/s12963-025-00369-x (PMC11874636; doi:10.1186/s12963-025-00369-x)
Supplement: Supplementary file 1 — Supplementary Material 1. [file 12963_2025_369_MOESM1_ESM.pdf]

Supplementary Information

A demographic assessment of the impact of the war in the Gaza Strip on the mortality of children and their parents in 2023

Benjamin-Samuel Schlüter
University of Toronto, Canada

Bruno Masquelier
UCLouvain, Belgium

Zeina Jamaluddine
London School of Hygiene and Tropical Medicine, UK

January 3, 2025

1 Stochasticity in war-related deaths

We had access to a list from the Palestinian Ministry of Health of 13,101 deaths that occurred between October 7 and December 31, with individual information on each death. We used information on the dates of birth and death, and sex of the deceased to compute the distribution of war-related deaths by sex and age. We assumed that this age- and sex-specific death distribution reflects the distribution of all deaths due to the war before December 31st and used it to redistribute the 21,822 deaths associated with the conflict by age and sex. This approach resulted in high stochasticity in the death counts and unusual peaks at ages 24 and 25, as illustrated in Figure S1a. To reduce the noise and potential age heaping, we aggregated death counts into 5-year age bins (Figure S1b). To match the age granularity from the life table (1-year age groups until 95+ years

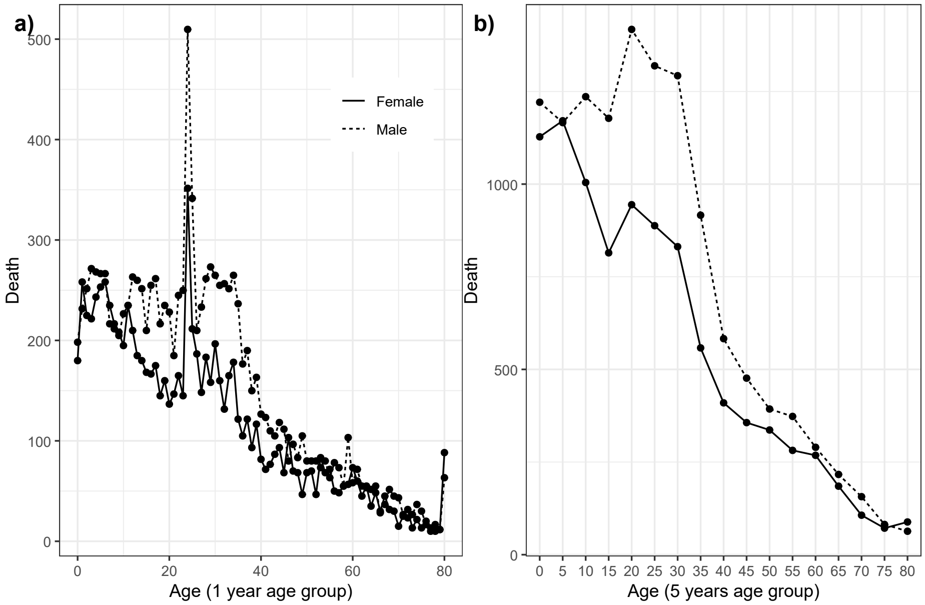

Figure S1: *(a) Death counts associated to the war up to the 31st December, (a) by 1-year age group (b) by 5-year age group.*

old), we ungrouped the death counts with the method proposed by Rizzi et al. (2015). Death counts by 1-year age group from the ungrouping method are shown in Figure S2, with the raw death counts also plotted for comparison. The death counts for single years of age were then combined with population exposures to compute sex- and age-specific mortality rates due to the war.

2 Stable population assumption in the kinship matrix model

In order to obtain the most reasonable estimates of the number of living parents per child less than 18 years old in 2023, we started the projection in 1997. In that year, an individual could be any age between 0 and 95 years old. As it is commonly done in these models (Alburez-Gutierrez et al. 2023), before 1997,

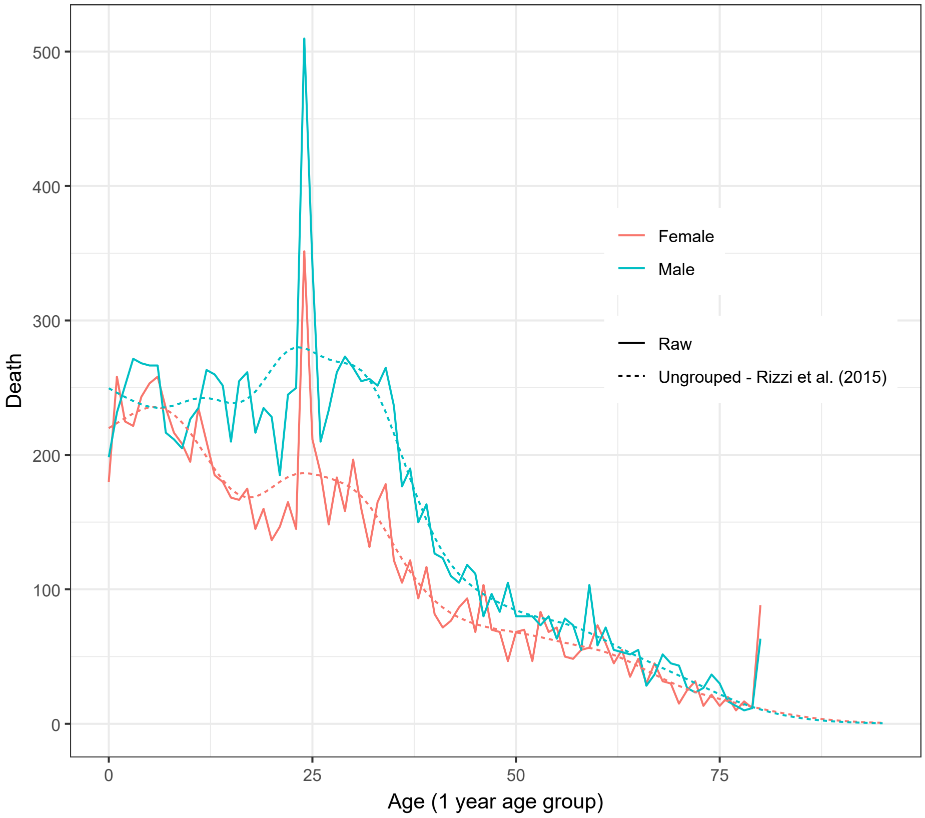

Figure S2: *Death counts by 1-year age group obtained by applying Rizzi et al. (2015) method, compared with the raw death counts.*

we assumed that the earliest available vital rates (mortality and fertility) have been operating for a long time (stable population assumption).

3 Monte-Carlo simulation and background life tables

The kinship matrix model being deterministic, the model outputs are not associated with uncertainty measures. To incorporate uncertainty into our estimates, we performed the projection within a Monte-Carlo simulation, running the pro-

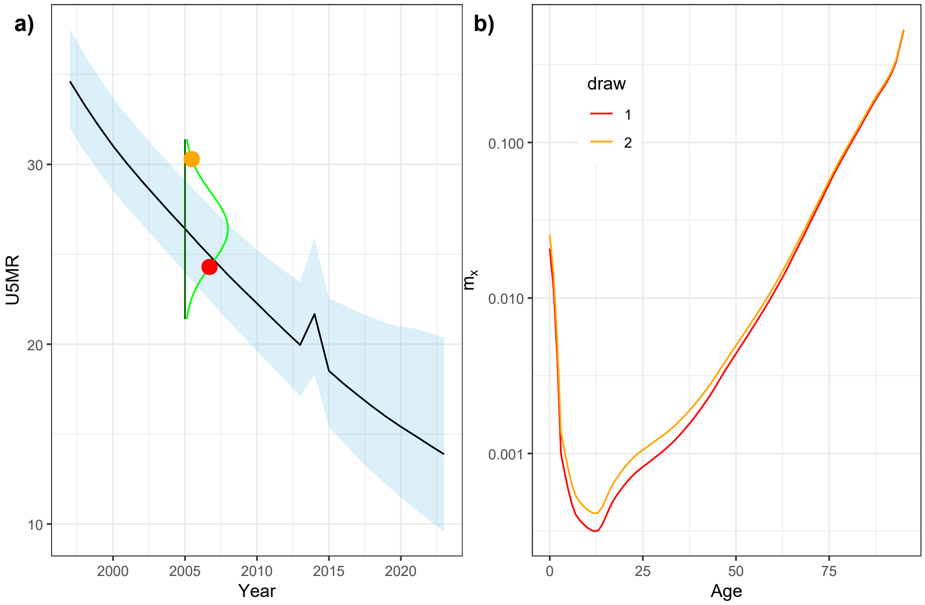

Figure S3: *Generation of background life tables in the Monte-Carlo simulation.*

jection over 2,000 iterations. At each iteration, uncertainty was incorporated through two elements: 1) uncertainty in the background U5MR estimates from the UN IGME and 2) stochasticity from the additional deaths due to the war. More precisely, at each iteration, we drew a U5MR estimate, assuming they are normally distributed using the 90% bounds provided by UN IGME. Figure S3a shows two U5MR draws for the year 2005. We then generated full life tables from the obtained U5MRs in 2005, using the West pattern of Princeton model life tables (Coale et al. 1983), as shown in Figure S3b.

We also reflected the stochasticity in war deaths by assuming that these deaths are Poisson distributed as follows,

$$ {}_1D^{war}_{x,s,i} \sim \text{Poisson}({}_1m^{war}_{x,s} \cdot {}_1E_{x,s}) $$

where ${}_1D^{war}_{x,s,i}$ corresponds to death due to the war, x is age, s is sex, i is the Monte-Carlo iteration, and ${}_1E_{x,s}$ is the population exposed to the risk. This

allowed us to generate age- and sex-specific death counts at each iteration. At each iteration, these death counts were then converted into mortality rates due to the war using the population exposures.

References

Diego Alburez-Gutierrez, Iván Williams, and Hal Caswell. Projections of human kinship for all countries. *Proceedings of the National Academy of Sciences*, 120(52):e2315722120, 2023.

Ansley J Coale, Paul Demeny, and Barbara Vaughan. *Regional model life tables and stable populations: studies in population.* Academic Press, New York, 1983.

Silvia Rizzi, Jutta Gampe, and Paul HC Eilers. Efficient estimation of smooth distributions from coarsely grouped data. *American Journal of Epidemiology*, 182(2):138–147, 2015. doi: 10.1093/aje/kwv020.
